# Beyond Circannual Fattening: Behavioural Flexibility and Sex‐Specific Strategies Enable Coping With High‐Elevation Winters

**DOI:** 10.1002/ece3.73482

**Published:** 2026-04-17

**Authors:** Sebastian Dirren, Carole Niffenegger, Fränzi Korner‐Nievergelt

**Affiliations:** ^1^ Swiss Ornithological Institute Sempach Switzerland; ^2^ Landscape Ecology and Ecosystems Conservation, Department of Environmental Sciencel University of Basel Basel Switzerland

**Keywords:** behaviour, high elevation, *Montifringilla nivalis*, physiology, snowfinch, winter

## Abstract

Winter survival at high elevations imposes severe physiological and behavioural constraints on resident birds, necessitating specialised adaptations to cope with low temperatures, high energy demands, and limited food availability. We studied physiological and behavioural strategies of the White‐winged Snowfinch ( *Montifringilla nivalis nivalis* ), a small passerine that inhabits high mountains year‐round. Using GPS tracking and morphological measurements, we examined seasonal changes in body mass, fat reserves, muscle mass, and lean body mass, alongside movement behaviour. Our results showed that snowfinches maintain larger fat reserves than overwintering lowland passerines, with fat accumulation regulated mainly by a circannual programme, likely reflecting a physiological adaptation to the high‐elevation environment. Seasonal changes in behaviour, including sex‐specific strategies and flexible movement patterns, appear to play an additional key role in coping with winter challenges in high mountains. Analysis of how temperature affects body mass indicated that fat accumulation is determined mainly by long‐term rather than short‐term fluctuations. These findings suggest that ongoing climate change, characterised by rising mean temperatures and more frequent extreme events, could disrupt this preparatory strategy, leading to reduced fat reserves and more frequent exposure to adverse winter and spring conditions.

## Graphical abstract

Winter at high elevations challenges resident species such as the White‐winged Snowfinch. Circannual‐regulated fat accumulation, along with flexible and sex‐specific movement strategies, likely play a critical role in coping with cold temperatures and unpredictable food, but may be vulnerable to climate change.

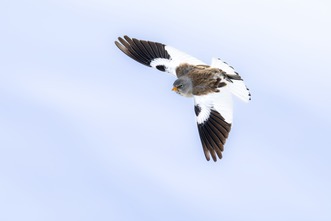

## 1 Introduction

Organisms enhance their survival and reproductive fitness by adjusting morphological, physiological, and behavioural traits in response to changing environmental conditions. Reversible adjustments that occur within individuals are referred to as phenotypic flexibility (Forsman 2015; Piersma and Drent 2003), a subcategory of phenotypic plasticity. This mechanism enables individuals to cope with seasonal or short‐term environmental variation through non‐genetic, reversible trait modifications. For example, the Alpine marmot *Marmota marmota* accumulates fat reserves, regulates down its metabolism and stays in its underground burrow for 6–7 months to endure the winter months (Ortmann and Heldmaier 2000; Ruf et al. 2023). Although morphology, physiology and behaviour are modified separately, there is a high degree of interdependence among them. Migratory birds for example, display hyperphagia before departure (i.e., behaviour), most likely induced by changes in hormone excretion (i.e., physiology), which leads to an accumulation of fat (i.e., morphology), which in turn will be needed as fuel for the higher metabolic requirements (i.e., physiology) during migration flights (Eikenaar 2017; McWilliams et al. 2004). Knowing about such mechanisms enables us to make interpretations beyond the traits for which changes are observed. In a migrating bird, estimating fat reserves allows predictions about its behaviour, such as readiness to depart and expected duration of stay at the site (Eikenaar and Schläfke 2013), as well as about its physiology, such as the distance the bird could fly with its fuel (Alerstam and Lindström 1990; Jenni and Jenni‐Eiermann 1987).

In resident bird species of temperate habitats, phenotypic flexibility follows a seasonal pattern, with individuals exhibiting reversible changes in morphology (Liknes and Swanson 2011), physiology (Petit et al. 2014), and behaviour (Pakanen et al. 2018) between summer and winter conditions. At higher latitudes and elevations, reduced food availability due to shorter day lengths, food scarcity, and lower temperatures in winter are among the key drivers of seasonal phenotypic flexibility (Williams et al. 2017). Energy storage in the form of lipids in birds represents a clear example of a flexible trait. While fat is the primary energy storage in birds (Guglielmo 2018; Jenni and Jenni‐Eiermann 1998; McWilliams et al. 2004), it is not only used to fuel endurance flights (Bairlein 2002; Guglielmo 2018) but also as an energy reserve for maintaining basal body functions such as thermoregulation (Gosler 2002; Lehikoinen 1987). Increasing fat stores is therefore a common strategy in birds to reduce the risk of starvation and death during winter.

The phenomenon of winter fattening has long been observed and reported in many resident bird species of temperate zones (Blem 1976; Dawson and Marsh 1986; Gosler 1996; King and Farner 1966; Lehikoinen 1987; Witter and Cuthill 1993). Although it is well‐known that fat accumulation generally begins in autumn, peaks in winter, and decreases towards spring, knowledge about the underlying mechanisms regulating this process remains limited for many species. The following two mechanisms could potentially explain these empirical observations. The first mechanism involves regulation by internal biological clocks, i.e., self‐sustained endogenous systems, that facilitate recurring life cycle stages in alignment with circannual rhythms, with day length as the primary timekeeping cue (Meddle et al. 2002; Rani and Kumar 2012). It has the character of an evolutionarily fixed program with annual periodicity, largely unaffected by short‐term fluctuations in environmental factors. If this mechanism is mainly responsible for the observed winter fattening, seasonal life cycle stages would be expected to exhibit distinct physiological traits shaping fat reserve dynamics. The winter fattening of the Svalbard rock ptarmigan *Lagopus mutus hyperboreus*, for example, has been shown to be controlled by a seasonal program, which depends on the annual changes in day length (Stokkan et al. 1995). The second mechanism operates on a shorter time scale, linking short‐term environmental changes, e.g., between consecutive days, directly to trait modifications. If winter fattening can be mainly explained by this mechanism, we would expect that fat accumulation is a result of short‐term response to proximate environmental factors, such as weather conditions, and food availability. In the great tit *Parus major* , effects on fat reserves could be attributed mainly to current and recent temperatures, and no underlying seasonal program has been found (Gosler 2002).

Since seasonality and extreme weather events are more pronounced towards higher elevational zones, fat accumulation is expected to be particularly important in high‐elevation specialists. The high‐elevation habitat poses characteristic challenges to its inhabitants. Abiotic factors like low temperatures and low oxygen partial pressure (Dillon 2006), high amounts of snow and a long period of snow cover (Hock et al. 2019; Keyser et al. 2023; Pauli et al. 2013), strong winds, and high solar radiation (Blumthaler et al. 1997) are considered the main selective forces for life above the tree line. Regarding food availability, resources are typically abundant in summer (Antor 1994; Brodmann and Reyer 1999), whereas in winter food supply becomes a limiting factor. Only a handful of bird species are thus able to inhabit these high‐elevation zones throughout the entire year. Most of the mountain bird species leave their breeding areas to spend the non‐breeding season under more favorable conditions at lower elevations (de Zwaan et al. 2022).

**FIGURE 1:**
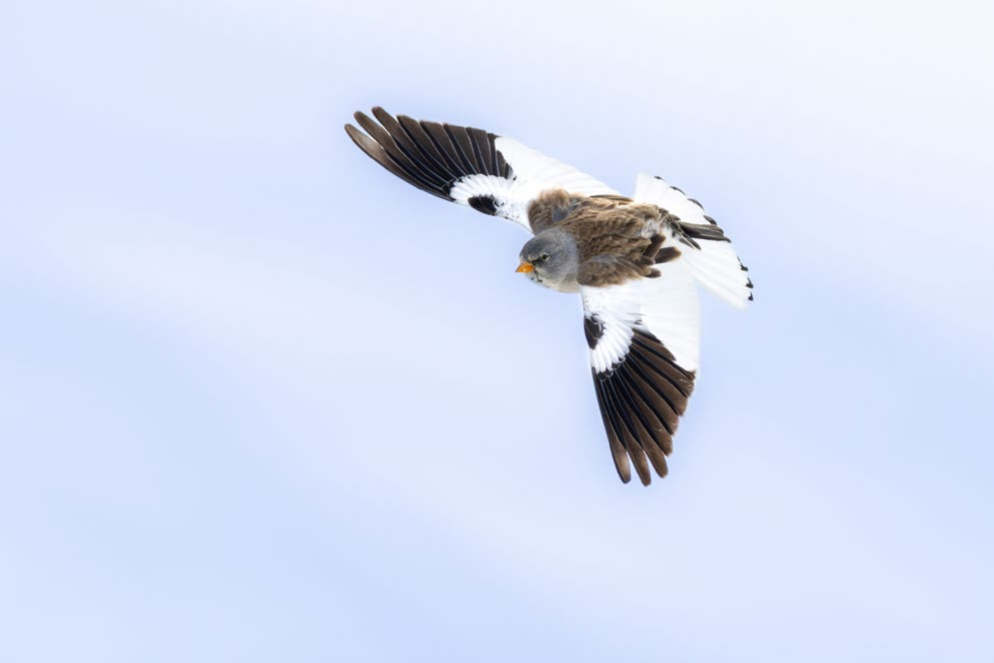
The White‐winged Snowfinch ( *Montifringilla nivalis nivalis* ) is a high‐elevation specialist. The species inhabits mountain ranges in southern and central Europe and occurs year‐round above the tree line. Photograph by Nino Jauch.

One of the specialist species capable of residing year‐round in alpine habitats is the White‐winged Snowfinch *Montifringilla nivalis nivalis* , hereafter referred to as snowfinch (Figure 1). This small passerine bird inhabits the subalpine, alpine, and nival zones of mountain ranges in southern and central Europe. In winter, snowfinches primarily feed on seeds from alpine plant (Wehrle 1989). In addition to the natural foraging grounds, local populations have learned to make use of anthropogenic food sources, e.g., visiting bird feeders. During winter, irregular heavy snowfall creates highly ephemeral food availability across both time and space. Depending on the amount of freshly fallen snow, wind, and avalanche activity natural foraging grounds become accessible or inaccessible within hours or even minutes. The high‐elevation habitat is thus characterised by highly unpredictable abiotic conditions.

Birds overwintering in high‐alpine environments are generally well‐adapted to cope with adverse winter conditions (Heiniger 1991b; Laiolo et al. 2014). However, even well‐adapted species, including the snowfinch, experience high mortality during the coldest months (Strinella et al. 2020; Williams et al. 2015). Winter survival thus has a substantial impact on population dynamics (Ozgul et al. 2010), and survival rates often differ between sexes (Payevsky 2021). Sex‐specific survival has been reported in alpine species such as the Alpine chough *Pyrrhocorax graculus* (Chiffard et al. 2019) and the snowfinch (Strinella et al. 2020). In both species, apparent survival was influenced by climatic factors. Female Alpine choughs showed lower apparent survival rates than males in spring following warm winters (Chiffard et al. 2019), and the annual apparent survival of female, but not male, snowfinches was negatively affected by hot and dry summers (Strinella et al. 2020). The mechanisms underlying these climatic effects on apparent survival remain unknown. Altered environmental conditions are primarily mitigated by morphological, physiological, and behavioural modifications before they become lethal. Therefore, studying the direct effects of environmental factors on morphology, physiology, and behaviour may help identify the underlying mechanisms by which changing environmental conditions influence demography.

We propose four main hypotheses regarding phenotypic flexibility as adaptation to survive winter at high elevations in snowfinches. First, we hypothesise that snowfinches exhibit seasonal phenotypic flexibility in morphological, physiological, and behavioural traits. Second, we hypothesise that this phenotypic flexibility is sex‐specific particularly in their movements (i.e., behaviour) and fat accumulation (i.e., morphology and physiology). Third, we hypothesise that the unpredictable environment experienced by snowfinches requires fat accumulation to be regulated not only in the short term but also in a circannual manner, mediated through distinct life cycle stages. If correct, short‐term body mass dynamics should differ between seasons, reflecting distinct physiological characteristics associated with different life cycle stages. Finally, we hypothesise that this short‐term body mass dynamics differ between the sexes, revealing sex‐specific and season‐dependent physiological capacities to cope with adverse winter conditions. To assess our hypotheses, we (i) examined a behavioural aspect of snowfinches by investigating movement patterns and home ranges using lightweight GPS tags. This revealed the year‐round occurrence of the species and highlighted sex‐specific behavioural patterns. Secondly, (ii) we focused on seasonal and sex‐specific morphological modifications, specifically in fat, muscle, and body mass. This shed light on morphological modifications driven by changing environmental conditions on a seasonal basis. Finally, (iii) we examined body mass dynamics of snowfinches as a short‐term response to changes in an abiotic environmental factor, namely temperature. This provided mechanistic insights into the relationship between body mass and ambient temperature.

## 2 Methods

### 2.1 Morphometrics in Winter

**FIGURE 2:**
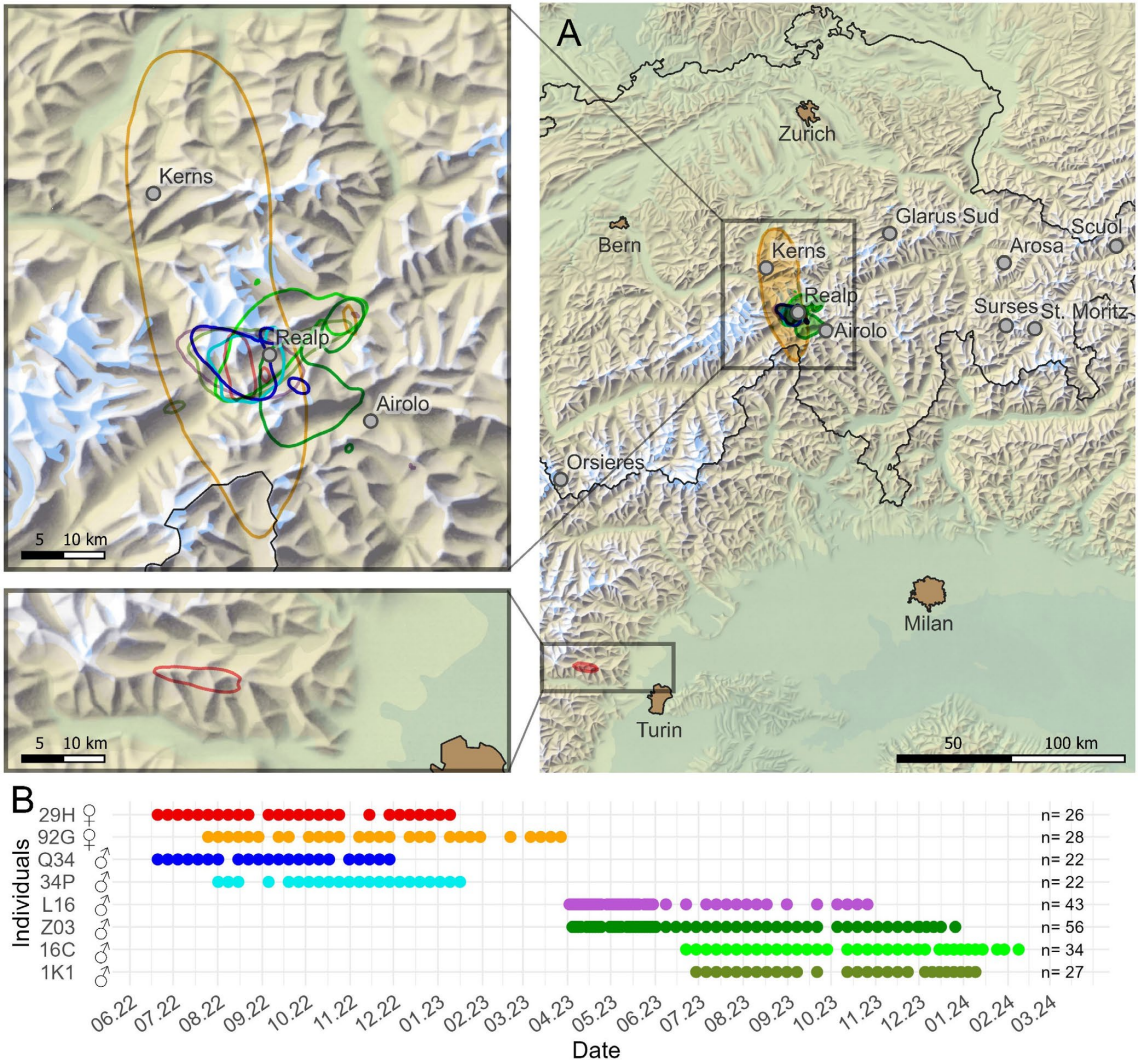
(A) Map showing the nine capturing sites (grey dots) in Switzerland. Coloured lines represent the 95% home range contours of eight snowfinches (colours match the individuals in B). Insets on the left show higher‐magnification views of the selected area. (B) Timeline of GPS locations (high‐quality fixes only) for the eight individuals. Dates indicate the first day of each month. Hillshade map swisstopo.

Snowfinches were captured in the winters 2017/18 to 2023/24 at bird feeders at nine study sites in the Swiss Alps (Figure 2A; Tables S1 and S2). All feeders were already established before our study began, i.e., often even decades ago, and maintained by local residents. They were located at houses in mountain villages, at lift stations in ski resorts, and in one case (i.e., Realp UR) at a hotel. Capture locations were chosen based on frequent observations of snowfinches. Snowfinches were fed with hemp seeds and sunflower seeds. In general, the feeders were supplied continuously from late autumn to spring, but the exact quantity of food and schedule of replenishment were unknown. As these were private sites, we were unable to control quantity and frequency of provided food, except on the days when we captured snowfinches and maintained the feeders ourselves. During those days, food was provided quasi ad libitum (i.e., it was continuously replenished and arranged so that many birds could access it at the same time). Birds were randomly captured between 07:00 and 18:00 on different days, either with white mist nets or with a whoosh net. After capturing, birds were immediately released from the net and placed in cotton bags until they were ringed and measured. The entire procedure lasted no longer than 1 h. All captured birds were tagged with the aluminium ring from the Swiss Ringing Scheme. The standard ringing protocol included measuring body mass, tarsus, wing chord, length of primary feather P8, fat and muscle scores (following Bairlein et al. 1995; Kaiser 1993) and collecting a saliva sample. Saliva samples were taken with a sterile cotton swap, transferred to a sample collection tube that contained 98% EtOH and stored at −18°C until further processing.

### 2.2 Genetic Sexing

DNA was extracted from the salivary swabs using the DNeasy Blood & Tissue Kit (Qiagen) according to the manufacturer's instructions, with the final elution volume reduced to 50 μL to increase DNA concentration. Molecular sexing was carried out using the primers ATP5A1‐F2 and ATP5A1‐R1 (Bantock et al. 2008), specifically designed for bunting museum samples and thus suitable for fragmented DNA. All PCRs were performed in the Eppendorf Vapo.Protect Thermal Cycler (Eppendorf). The 20‐μL reaction mix contained 2 μL of DNA, 0.4 μM of each primer, 0.2 mM of each dNTPs, 2 μL 10xPCR Buffer, 3 mM MgCl_2_ and 2.5 U of Taq DNA Polymerase (Qiagen). Cycling conditions consisted of initial denaturation at 94°C for 5 min, followed by 40 cycles of 94°C for 30 s, 50°C–48°C touch‐down for 30 s, 72°C for 30 s, with a final extension at 72°C for 10 min, with the lid heating on 105°C. Negative controls were included in each set of PCRs. Following amplification, the PCR products were visualised on 3% agarose gel, stained by GelRed 10′000× (Biotium). Presence of two bands was scored as female (ZW) and presence of one band as male (ZZ) genotype.

### 2.3 GPS Tracking and Home Ranges

In addition to the winter capturing, we captured birds in the summers 2022 and 2023 at one breeding site (i.e., Realp UR, 2430 m a.s.l., see Table S1), with a net at the entrance of nest boxes. We deployed Lotek PinPoint 10 GPS loggers either at this breeding site or at our winter capturing site in Realp (i.e., locations ~4 km apart) to obtain high‐precision location data from tagged birds over a period of several months. GPS tags were fitted to the birds with a leg‐loop harness. The weight of the tag plus harness was ~1.5 g, which corresponds to less than 5% of the bird's weight. In 2022, we deployed nine loggers during the breeding season, with locations always collected at 08:00 UTC on the following schedule: weekly from June 2022 to March 2023 and every other day from April to May 2023. In 2023, we deployed 10 GPS loggers (four in April and six during the breeding season), and locations were collected at the following intervals: every other day from April to May 2023, weekly from June to November 2023, and every fifth day from December 2023 to February 2024. We first filtered the fixes to retain only high‐precision locations, keeping those that were obtained with at least four satellites and had a Horizontal Dilution of Precision (HDOP) value ≤ 10. Analysis of data collected from 10 PinPoint tags under realistic conditions (i.e., along transects at two study sites: Realp and Kerns) revealed that, after applying this quality filtering, the mean horizontal positional deviation was 19.5 m (SD = 38.3 m, max = 483 m, *n* = 495). Instead of using the GPS‐derived elevations (mean vertical positional deviation = ~100 m), we projected the high‐quality locations onto the EuroDEM model (EuroGeographics) to obtain elevations with a mean vertical positional deviation of ~10 m, compared to the high‐precision elevation model swissALTI3D (Federal Office of Topography swisstopo). Locations of individuals were used to estimate individual home ranges using kernel density estimations (KDE). For one individual (29H), which had two GPS point clusters separated by 180 km, we calculated separate home ranges for each cluster and merged them afterwards. The KDE were calculated with the ctmm package in the R environment (Calabrese et al. 2016). Assuming the collected positions are not autocorrelated, we applied KDE with the independent identically distributed (IID) model. To assess how the spatial distribution of the feeders (i.e., at our study sites) and potential anthropogenic food sources around buildings influence snowfinch occurrence patterns, we extracted all GPS points within a 50 m radius of buildings. For locations in Switzerland, we used the swissBUILDINGS^3D^ 2.0 layer provided by the Federal Office of Topography swisstopo, while for Italy, satellite imagery from Google Earth was used to manually verify building locations. Additionally, we checked if these locations were within a 50 m radius of feeders at our study sites. All data analysis, plotting and illustration was conducted using R 4.4.3 (R Core Team 2021) and the QGIS software Version 3.34.10 (QGIS Development Team 2009).

### 2.4 Temperature Trends and Day Length

Temperature measurements were provided by MeteoSwiss, the Swiss Federal Office of Meteorology and Climatology. We selected four meteorological stations two in the eastern part of Switzerland (i.e., Weissfluhjoch, 2691 m a.s.l., and Segl‐Maria, 1804 m a.s.l.), and two in the central part of the Swiss Alps (i.e., Titlis, 3044 m a.s.l., and Andermatt, 1434 m a.s.l.). We combined data from two stations at different elevations within each region by calculating the mean values of their temperature measurements. The stations covered the elevational range predominantly inhabited by snowfinches. Therefore, we considered the mean values from these stations biologically relevant to the species. Subsequently, we associated the temperature means of the day before capture to the respective birds of the corresponding region (i.e., eastern region: Arosa, Scuol, St. Moritz and Surses; central region: Kerns, Glarus Süd, Realp, Airolo and Orsières). To calculate the capture time since sunrise and day length we used data from Time and Date AS (https://www.timeanddate.de). We extracted sunrise times for the study period from December 2017 to April 2024 at the location 46°37′16.7″N, 9°52′22.0″E.

Trends in long‐term average temperatures for the standard 30‐year climatological period 1991–2020, from December to April, were comparable between the weather stations Segl Maria (SIA) and Weissfluhjoch (WFJ), which differ in elevation by 887 m (Figure S1). On average, temperatures at the high‐elevation station were 2.9°C lower. Temperature followed a seasonal pattern with lowest temperatures from December to February (with January being the coldest at SIA and February at WFJ), followed by a marked temperature rise at both stations. The temperature difference between the stations was smallest in January (1°C) and largest in April (5.2°C). The combined long‐term average temperatures of the two stations (i.e., Long‐term Temp. SIA/WFJ) can be considered a long‐term abiotic factor affecting snowfinches in the Swiss Alps.

We compared these long‐term trends with actual temperatures recorded during the period of our study (Dec 2017–Apr 2024). We calculated the mean temperatures for this period at SIA/WFJ and Andermatt (ANT)/Titlis (TIT), respectively (Figure S1). The mean temperatures of SIA/WFJ and ANT/TIT were highly correlated across all months, suggesting that the mean temperatures are comparable between our study regions. A comparison of the SIA/WFJ mean temperatures from 2017 to 2024 with the combined SIA/WFJ long‐term average temperatures revealed that December, January, March, and April were highly similar to the long‐term average (mean difference ± SD: 0.38°C ± 0.14°C), whereas February was 2°C warmer during the study period compared to the long‐term average of this month (Figure S1).

While the increase in day length between December and January is only 25 min, it rises by 1 h 17 min between January and February, 1 h 36 min between February and March, and 1 h 40 min between March and April (Figure S1).

### 2.5 Seasonal Dynamics of Body Mass, Fat Mass, Muscle Mass, and Lean Mass

To convert each individual's fat and muscle scores into grams we used a linear model with body mass (in g) as response variable and fat and muscle score as predictors (Salewski et al. 2009). The coefficients of that model measure how much, in average, body mass increases by an increase of one fat or one muscle score. We interpret that increase as the average change of fat and muscle in gram, respectively, that corresponds to one score of fat or muscle. Wing chord has been shown to be the best general measure of body size in small passerines (Gosler et al. 1998), therefore we included wing chord to correct for size differences as a covariate in the model. We also included the interactions of wing chord with both fat and muscle scores because an increase of one fat or muscle score may be associated with a higher increase in fat or muscle mass in larger birds compared to smaller ones. Sample size was 1690 measurements of 1386 Individuals in 7 years. From the model, we derived the relationship between mass per unit of fat and muscle score (i.e., the coefficients for fat and muscle) and wing chord. This allowed us to calculate for each individual fat mass and muscle mass based on its wing chord and the respective scores.

Subsequently, we analysed how body mass, fat mass, and muscle mass varied with month and time of day for each sex by fitting three separate linear mixed‐effects models, one for each response variable. In other words, we used measurements collected at different times of day to model average values of body mass, fat mass, and muscle mass for each sex and month (December to April). Including time of day as a covariate allowed us to account for differences in capture times and to estimate hourly gain rates for each response variable. Specifically, we calculated average values for males and females 4 h after sunrise for each month. Hourly gain rates were also calculated for each sex and month, representing average increases or decreases in the response variables across individuals. Our analyses thus reflect population‐level trends rather than changes within individual birds.

Month was used as a categorical predictor (five levels: December to April). Time of the day was included as a continuous covariate and measured as hours since sunrise. Expressing time relative to sunrise, rather than clock hour, provided a biologically meaningful variable representing the foraging time available prior to capture. We further included sex as a predictor and all two‐way interactions, i.e., sex and month (allowing sex‐specific effects to vary by month), sex and time since sunrise (allowing sex‐specific gain rates) and time since sunrise and month (allowing gain rates to vary across months). Day nested within location, year and individual were included as random effects in all three models, and the observer (person who measured the bird) was additionally included as random effect in the models for fat and muscle mass to account for among‐observer variance in assigning fat and muscle scores. Sample sizes were 1776 measurements of 1426 individuals for body mass, 1690 measurements of 1386 individuals for fat and muscle masses. All models were fitted by the function lmer from the package lme4 (Bates et al. 2015). Goodness of fit was assessed visually based on the standard diagnostic residual plots. Monthly lean body mass was calculated by subtracting average fat and muscle masses from the average total body mass for each month.

Assuming that, on average, daytime fat accumulation equals nighttime fat loss, we estimated the fat requirements of snowfinches during winter nights. Daily fat accumulation can be calculated by multiplying the winter average of the hourly fat gain rates by the available foraging time of 10.3 h (average day length in winter plus 1 h; personal observation). We then compared these values with the average winter fat masses to estimate the number of nights until complete fat reserve depletion.

### 2.6 Short‐Term Effects of Temperature on Body Mass

Because body mass measurements are more precise than fat mass estimates, we used body mass as the response variable in a model for analysing short‐term temperature effects on body mass. In the short term, changes in body mass basically reflect changes in fat mass (Lehikoinen 1987). We modelled the average body mass dependent on the temperature on the day before a bird was captured and the time since sunrise. For this analysis, we distinguished between two seasons: winter (December to February) and early spring (March and April). The model included temperature as an environmental predictor. Wing chord was included to correct for size differences. Furthermore, month, time since sunrise and sex were included as predictors of the model. We included the two‐way interactions: time since sunrise and sex (allowing sex‐specific body mass gain rates), temperature and sex (allowing sex‐specific temperature effects), temperature and season (allowing temperature effects to vary between seasons), sex and season (allowing sex‐specific seasonal effects), and the three‐way interaction season and temperature and sex (allowing sex‐specific temperature effects to differ between seasons). Day nested within location and year were included as random factors in the model to account for non‐independence of the measurements taken on the same day, at the same location and within the same winter. Analogous to the analyses of seasonal dynamics in body mass, fat mass, and muscle mass, the modelled short‐term effects of temperature on body mass reflect population‐level patterns rather than changes within individual birds. Sample size was 1690 measurements of 1386 individuals. The model was fitted with the brm function from the brms package version 2.22.0 (Bürkner 2017). We used the default weakly informative priors and four Markov chains with 2′000 iterations. The convergence of the Markov chains was visually assessed using trace plots and with the Gelman‐Rubin Statistic (*Ȓ* < 1.01).

## 3 Results

### 3.1 GPS Tracking and Home Ranges

In total we recaptured 9 out of 19 tagged birds and retrieved their GPS loggers. While data were successfully downloaded from 8 loggers (two females/six males), data retrieval failed for one logger due to water ingress. The 8 tags collected a total of 289 valid GPS fixes, while 17 attempts failed due to insufficient satellite coverage. Quality filtering resulted in 258 locations from 8 individuals (Figure S2A–H). We collected location data from four individuals between June 2022 and March 2023, and from another four individuals between April 2023 and March 2024 (Figure 2B). Elevations (in m a.s.l.) during winter were on average 408 m lower than during the rest of the year (December—February: min = 1048 m, mean = 2127 m, max = 2955 m, *n* = 45; March—November: min = 1458 m, mean = 2534 m, max = 3122 m, *n* = 213; Table S3). Elevational distribution differed slightly among individuals with the elevational range between 2000 and 3000 m a.s.l. being predominantly occupied by all individuals (Figure S2I). The home ranges were markedly different between males and females (Figure 2A and Figure S2A–H). While the six males utilised home ranges of comparable size (57.6–125.3 km^2^), one female (92G) utilised an area that was at least 6.5 times larger (835.5 km^2^), and the other female (29H) had a discontinuous home range consisting of two areas ~180 km distant from each other (Figure 2A and Figure S2A–H; Table S3). Locations logged in winter (Dec—Feb) were compared to those from other seasons for all individuals (Figure S2A–H). In general, no clear spatial patterns were observed between these two periods, except for one female (29H). All winter locations of this female were in the region near Turin, ~180 km southwest of its breeding site in Switzerland (Figure 2A and Figure S2F). Of 258 recorded locations, ten were within a 50 m radius of buildings (Figure S2A–H), including one within a 50 m radius of the feeder at the winter capturing site in Relap UR (Figure S2A–H and Table S1). The proportion of locations near buildings was higher in winter (5 of 45, 11%) than in summer (5 of 213, 2%).

### 3.2 Seasonal Dynamics of Body Mass, Fat Mass, Muscle Mass, and Lean Mass

**FIGURE 3:**
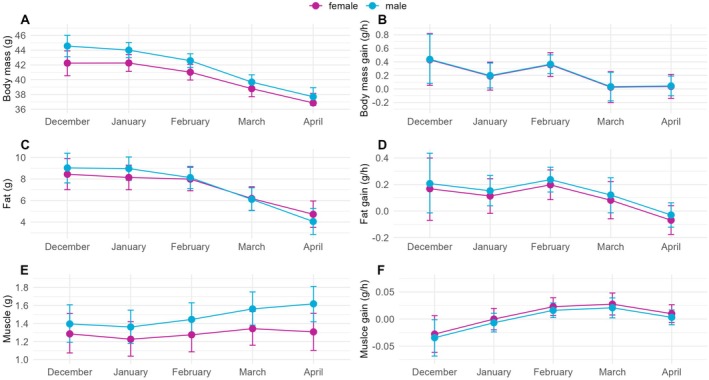
Seasonal dynamics of body mass, fat mass, and muscle mass modelled using linear mixed‐effects models. Error bars indicate the 95% confidence intervals. (A, C, E) show average masses of the respective body parts for males and females 4 h after sunrise. (B, D, F) depict the hourly gains for each body part. A pronounced decline in body mass and fat is observed between February and March.

Males and females had similar seasonal patterns of body mass. Average body mass was highest from December to February and showed a marked decrease between February and March and a further decrease between March and April (Figure 3A; Figure S3; Table S4). Females were consistently lighter than males, with the greatest absolute mass difference observed in December and the smallest in April (Figure 3A; Figure S3; Table S4). This difference in body mass can be mainly explained by the body size difference between the sexes. Males had an average wing chord of 123.5 mm (SD = 2.7, *n* = 1292), compared to 118.3 mm (SD = 2.6, *n* = 458) in females. Average hourly body mass gain rates were similar for males and females, with highest values in December and February, intermediate values in January and lowest in March and April (Figure 3B; Figure S3; Table S4).

The seasonal dynamics of fat mass were similar for both sexes and closely matched the body mass pattern, with highest average values for the months December to February and a subsequent decrease between February and April (Figure 3C; Figures S4 and S5; Tables S5 and S6). Body fat percentages were comparable between the sexes throughout winter and early spring, with females having slightly lower body fat in January and slightly higher values in March and April (Figure S6). Hourly fat gain rates followed a similar seasonal pattern in both sexes and largely mirrored the trends observed in body mass gain rates: highest in December and February, intermediate in January and March, and close to zero in April. However, unlike body mass gain rates, fat gain rates in March were nearly as high as those in January (Figure 3D; Figures S4 and S5; Tables S5 and S6). Based on winter averages of hourly fat gain rates, a daily fat accumulation of 1.6 g in females and 2.0 g in males was estimated, corresponding to the fat reserves required for the night. Given the average winter fat masses (females: 8.2 g; males: 8.7 g), these reserves would be completely depleted after 5.0 nights and 4.2 nights in females and males, respectively.

In contrast to body and fat masses, muscle mass showed different seasonal dynamics, with the lowest average values occurring in December and January, followed by an increase until March in both sexes, and a further increase in males but a decrease in females in April (Figure 3E; Figures S4 and S7; Tables S5 and S7). Males consistently had more muscle mass in absolute terms, but muscle mass as a percentage of body mass was similar between females and males throughout the months (Figure S6). Hourly muscle mass gain rates were highly similar between the sexes, close to zero in January and April, slightly negative in December and slightly positive in February and March (Figure 3F; Figures S4 and S7; Tables S5 and S7).

Variations in body mass were primarily driven by changes in fat mass and, to a lesser extent, in lean body mass (Figure S6). The highest average lean body masses were observed during winter, specifically in January for females and December for males, with the most prominent decrease estimated between January and February for females and between February and March for males.

While fat mass predominantly explained the observed variation in body mass, lean mass accounted for most of the remaining variation, whereas muscle mass contributed negligibly. In males, fat mass differed by up to 5 g (9.0 g in December versus 4.0 g in April), and in females by up to 3.7 g (8.4 g in December versus 4.7 g in April). Lean mass in males and females varied by a maximum of 2.1 g between December and March, and between January and April, respectively. In contrast, average muscle mass differed by only 0.4 g across all months, ranging from 1.2 g in January to 1.6 g in April (Figure S6).

### 3.3 Short‐Term Effects of Temperature on Body Mass

**FIGURE 4:**
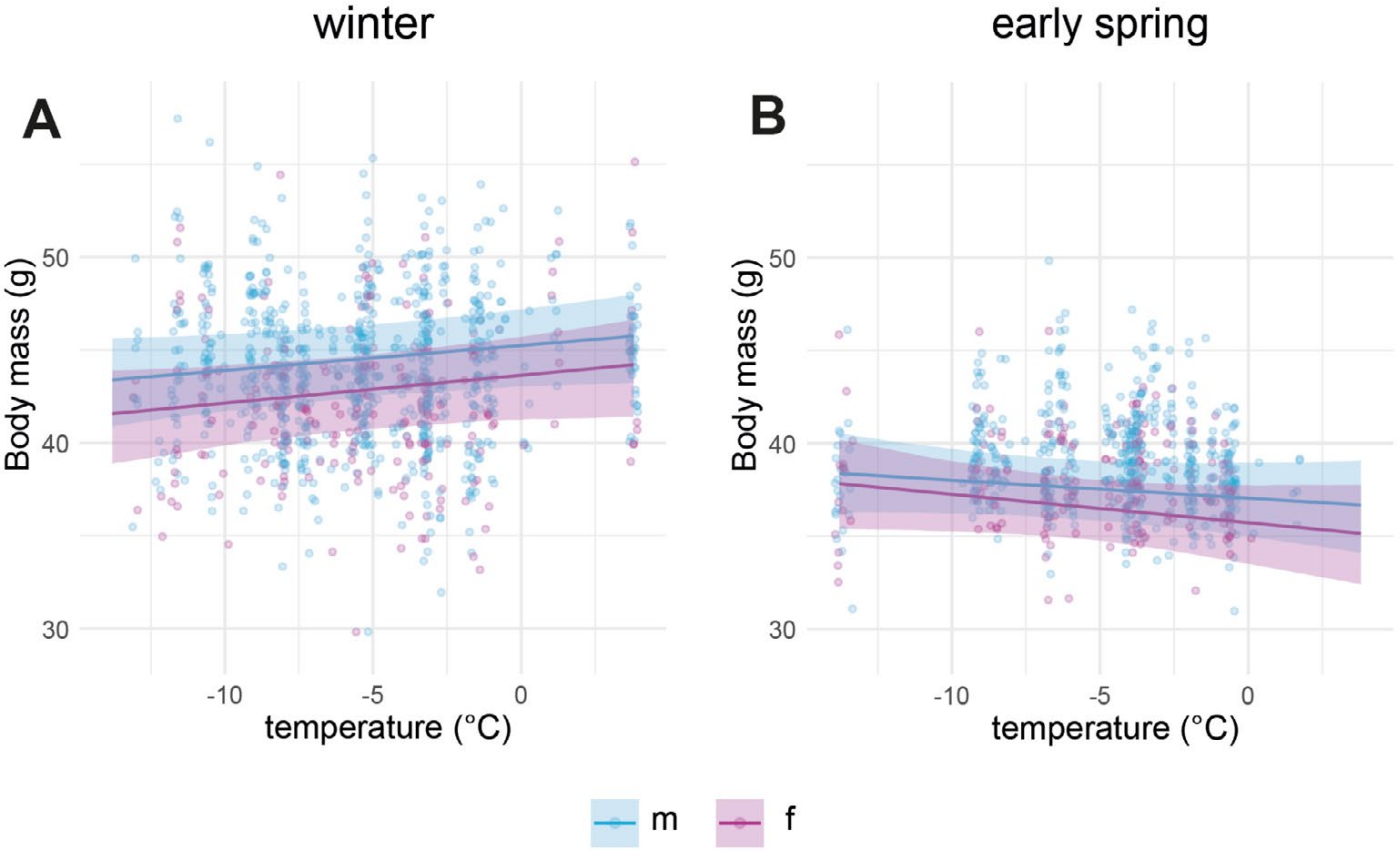
Average body mass in relation to the temperature on the day before capture in winter (A) and early spring (B). Points represent raw data, and shaded areas show the 95% credible interval from posterior predictions of the linear mixed‐effects model. All other model predictors were held at their mean values.

The relationship between the mean temperature during the day before a bird was captured and the body mass varied seasonally. From December to February, body mass was higher after days with higher temperature (mean male: 0.13 g °C^−1^, 95% CrI: −0.03–0.28 g °C^−1^; mean female: 0.15 g °C^−1^, 95% CrI: −0.02–0.32 g °C^−1^; Figure 4; Figure S8; Table S8), whereas from March to April, higher temperatures were associated with lower body mass on the next day, particularly for females (mean female: −0.15 g °C^−1^; 95% CI: −0.37–0.05 g °C^−1^; mean male: −0.10 g °C^−1^; 95% CI: −0.29–0.09 g °C^−1^; Figure 4; Figure S8; Table S8).

## 4 Discussion

### 4.1 Seasonal Flexibility in Behaviour: Movements, Home Ranges and Site Use

In line with previous studies (Heiniger 1991b, 1991a), our GPS data confirmed for eight individuals that snowfinches are capable of inhabiting high‐elevation habitats year‐round. All tracked individuals predominantly occupied the alpine and nival zones, with home ranges located within the main mountain range of the central Alps, except for one female (29H) that spent the winter in the southern Alps (Figure 2A).

In the central Alps, the snowfinch is a human‐associated species, albeit to a much lesser extent than its relative, the house sparrow *Passer domesticus* . Snowfinches exploit various anthropogenic food sources near buildings, but unlike house sparrows, they generally avoid dense settlements and access anthropogenic food mainly under unfavourable conditions in winter (personal observation). This pattern is reflected by the fact that the vast majority of GPS locations (96%) were recorded more than 50 m from buildings (Figure S2A–H), likely corresponding to natural feeding grounds or points recorded during commuting flights between sites, such as between food sources, roosting areas, and breeding sites. Consequently, features of the natural habitat, including feeding grounds, roosting areas, and breeding sites, may exert a stronger influence on movement patterns/home‐range use than anthropogenic food sources.

We found that snowfinches exhibit behavioural flexibility to adjust to winter conditions. This is consistent with previous studies that observed changes in snowfinch behaviour in response to harsh winter conditions, such as increased gregariousness with colder temperatures and higher precipitation (del Mar Delgado et al. 2021). Individuals showed an elevational shift towards lower elevations during winter. The elevational shift suggests that snowfinches altered their behaviour, likely movement patterns and/or the use of different sites, to adjust to the changing environmental conditions. Less snow at lower elevations may lead to more suitable feeding grounds at these sites during winter. In addition to the natural feeding grounds, anthropogenic food sources tend to be located at the bottom of the valleys, which together could explain the observed elevational shift. The higher proportion of GPS locations near buildings in winter (11%) compared to the rest of the year (2%) supports the conclusion that snowfinches used anthropogenic food sources more frequently in winter compared to the rest of the year, further demonstrating seasonal behavioural flexibility.

Previous studies based on genetic information and stable isotope analyses had already suggested that snowfinches exhibit partial and opportunistic migrations (Resano‐Mayor et al. 2017, 2020). Here, we provide the first direct evidence of migration in female snowfinches. One female (29H) undertook a short‐distance migration to an overwintering site and back, breeding again with the same male (Q34) as in the previous year. The other female (92G) remained in a broader area around the breeding site. The home range of this female was more than 6.5 times larger than those of all other tracked individuals, indicating an alternative behavioural strategy (Figure 2A; Figure S2A–H; Table S3). Larger home ranges may represent a strategy to increase survival probability under conditions of low resource availability (Mcloughlin and Ferguson 2000; Rolando 2002; Rühmann et al. 2019).

Home range sizes and movement patterns of the two females differed markedly from those of the six males, all of which occupied relatively restricted areas around the capture and/or breeding sites. Males may benefit from remaining in breeding areas either by increasing their chances of occupying high‐quality territories and nest sites as shown for other bird species (Morinay et al. 2024; Smith and Moore 2005), or by enhancing their opportunities to obtain mates (Kokko et al. 2006). The low proportions of captured females at the bird feeders (winter: 23.0%; early spring: 29.6%; Table S2) could thus reflect these behavioural differences. Partial migration and sex‐specific habitat use may contribute to the male‐dominated presence at artificial feeding sites. Although our GPS data are consistent with sex‐specific winter behaviours, the small and male‐biased dataset (i.e., six males, two females) limits quantitative population‐level assessment.

### 4.2 Seasonal Flexibility in Body Mass, Fat Mass, Muscle Mass, and Lean Body Mass

The optimal body mass concept posits that birds adjust their body mass according to a trade‐off between starvation risk and predation risk (Lima 1986). In this context, winter fattening illustrates phenotypic flexibility in body mass that is directly linked to the relative importance of starvation and predation risks. Predation pressure was not specifically assessed in this study, but predation risk generally decreases with elevation (Crouchet et al. 2024; Domínguez‐Godoy et al. 2020; Moreno‐Rueda et al. 2019), suggesting that high‐elevation specialists are exposed to lower predation, particularly during high winter. During this period, starvation risk is therefore likely to play a disproportionately larger role in the trade‐off compared to the relatively low and stable predation risk. Our observations of body and fat masses in snowfinches are consistent with the expected adjustment of body mass upon changed starvation risk. In high‐elevation habitats, starvation risk is elevated during winter due to scarcity and unpredictability of food sources, combined with higher energy demands. Consequently, birds are expected to have higher body mass through increased fat reserves. Indeed, snowfinches exhibited a clear seasonal phenotypic flexibility in body mass and fat reserves, with peak values in high winter and a marked decline between February and March (Figure 3A,C), consistent with a reduced starvation risk in early spring. The observed reduction in fat reserves is likely driven by increased accessibility to natural feeding grounds due to snowmelt, combined with lower energy demands for thermoregulation. The temporal pattern of winter fattening in this high‐elevation specialist is comparable to that observed in lowland passerine species (Blem 1976; Dawson and Marsh 1986; Lehikoinen 1987; Newton 1972).

However, the average winter body fat of about 20% in snowfinches appears to be at the upper limit of what has been reported for small passerines such as tits and finches at lower elevations (5%–25% of body mass see Dawson and Marsh 1986; Lehikoinen 1987). To assess safety margins in energy reserves, i.e., how long birds can survive on their fat stores, absolute fat mass must be considered relative to energy costs. Previous studies estimated overnight fat loss by comparing evening and morning fat masses. This loss was then related to absolute fat mass to assess how long birds could survive on their energy reserves. For small passerines overwintering at lower elevations, depletion of fat reserves was typically estimated to occur after 1–2 nights (Blem 1976; Dawson and Marsh 1986). In contrast, we estimated daily fat accumulation as an indirect measure of nighttime fat loss, assuming that, on average, daytime fat gain equals overnight fat loss (Lehikoinen 1987). We subsequently compared these values to winter fat reserves to estimate how many nights it would take for complete depletion. Both approaches provide theoretical values under the assumption of no food intake and average abiotic conditions, which may be unrealistic. Complete absence of foraging is likely very rare and only expected under extreme weather conditions for a short amount of time. Moreover, extreme weather events usually simultaneously increase overall energy demand, for example due to colder temperatures and stronger winds, which could result in substantially higher overnight fat requirements. Although the survival may be considerably lower under natural conditions, it allows a comparison between different species. For snowfinches, we estimated a survival of 4.2–5.0 nights with their winter fat reserves, which is substantially higher than estimates for overwintering small passerines at lower elevations. The harsh winter conditions, the high unpredictability of food, and the relatively low predation risk in alpine habitats are likely the main drivers of larger fat reserves, presumably as an adaptation in high‐elevation specialists. In addition to larger fat reserves, reduced energy expenditure likely contributes substantially to the larger safety margins. For example, snowfinches use specific roosting sites in rock cavities that provide favourable microclimates (Heiniger 1991b). Plumage with better insulation properties (Lei et al. 2002) may further reduce overall thermoregulatory costs.

Muscle mass followed the inverse pattern of fat mass, being lowest in the coldest months and rising towards early spring (Figure 3E). This contrasts with the commonly observed increase in muscle mass for cold acclimatisation in small avian species. Apart from being necessary for endurance flights, enlarged muscles enhance thermogenesis through muscle shivering (Carey and Dawson 1999; Petit et al. 2014; Swanson and Vézina 2015; Zheng et al. 2008). It is unclear why we observed the opposite pattern in snowfinches. Consistent with findings in other species (Barceló et al. 2016; Milbergue et al. 2018), muscle shivering may not be necessary to enhance thermogenesis in this high‐elevation specialist.

The higher lean body masses observed for snowfinches in winter align well with observations in other cold‐adapted bird species, where enlarged digestive and excretory organs contribute to increased lean mass in winter (Barceló et al. 2016). Similarly, for snowfinches, it may be important to adjust the sizes of the intestines and liver to accommodate increased food intake and turnover rates.

Taken together, seasonal patterns in body mass, fat mass, muscle mass, and lean body mass observed in this study suggest that phenotypic flexibility is expressed across all of these traits in both sexes. Seasonal variation in body mass was driven primarily by changes in fat mass and, to a lesser extent, by lean body mass, whereas muscle mass contributed relatively little. Overall, sexual size dimorphism largely accounts for the observed differences in body mass between the sexes, while the proportional contributions of the different body parts were highly similar in males and females across all months from December to April (Figure S6).

Since snowfinches were captured only at sites with anthropogenic food supply, our results may not represent individuals that do not visit feeders. The low proportion of females at feeders (Table S2) suggests that a considerable part of the female population moves away or uses alternative feeding grounds during winter and early spring. These may include natural foraging grounds as well as other sources of anthropogenic food, such as leftovers in mountain villages, or around restaurants. GPS tracking supports this, showing that females maintain larger home ranges and may migrate to overwintering sites farther from their breeding grounds than males. Individuals that did not visit our feeders, i.e., disproportionally more females, were not included in the analyses, therefore our sample of females may be biased towards individuals that resemble the males in behaviour and physiology. Different behaviour during winter is likely associated with different physiological and morphological characteristics. Therefore, the weight dynamics we observed cannot be directly extrapolated to the entire snowfinch population. On the other hand, mechanistic aspects, such as the regulation of fat accumulation and the effect of temperature on body mass, are likely characteristic of the species as a whole and may even be comparable to those of other high‐elevation specialists.

Individuals visited bird feeders primarily during adverse weather events, and at some sites, even exclusively (personal observations). These observations suggest that feeders can partially compensate for temporary declines in natural food availability. While visiting feeders reduces food unpredictability during adverse environmental conditions, the associated low temperatures still increase energy expenditure for thermoregulation. If we attribute higher energy costs in winter primarily to thermoregulatory costs, which are closely linked to ambient temperature, we expect body masses to correlate well with long‐term average monthly temperatures. Such a correlation suggests that birds are well adapted to the environmental conditions they have experienced over evolutionary time scales. Indeed, snowfinch body mass was negatively correlated with the monthly long‐term average temperature. The 30‐year average temperatures showed a marked increase between February and March (Figure S1), corresponding to the pronounced decrease in body mass and fat reserves observed in this study (Figure 3A,C). In contrast, a marked temperature increase was already observed between January and February during the study period (i.e., averaged over the years 2017–2024, see Figure S1), which was not reflected in the seasonal body mass and fat patterns (Figure 3A,C). It is possible that food predictability and/or predation risk have changed in recent years, with February now presenting more unfavourable conditions compared to the past, which could mask any potential reduction in fat reserves resulting from lower thermoregulatory costs. However, it is more likely that snowfinches simply did not respond to the drastic temperature increase over the past decades. This would be in line with our hypothesis that fat accumulation is regulated by a circannual program driven by an internal clock. A programme operating on an evolutionary timescale is expected to be more rigid, which could explain why the dynamics of fat accumulation aligned more closely with long‐term temperature trends than with short‐term deviations in recent years.

Temperatures have changed drastically over the past decades due to climate warming. In the Alps, the increase is about twice that of the global average, with Switzerland's mean temperature rising around 2°C since pre‐industrial times (Federal Office for the Environment et al. 2020). Rising temperatures may have caused a mismatch between the timing of fat depletion and the seasonal increase of temperature. We have already shown for snowfinches that the timing of breeding lags behind the advancing snowmelt (Schano et al. 2021), leading to increased temperature conditions during the breeding season (Niffenegger et al. 2025). This study now indicates that also during the non‐breeding season, abiotic factors are changing at a faster pace than snowfinches are adjusting their morphological and physiological traits.

### 4.3 Short‐Term Effects of Temperature on Body Mass

In field studies of small passerines, short‐term effects of ambient temperature have typically shown negative correlations between body mass and the mean or minimum temperature on the day of capture (Gosler 1996; Pilastro et al. 1995). This has been interpreted as a proximal effect of temperature, i.e., individuals sense current conditions to decide about how much fat to accumulate. It has already been recognised by King (1972) that a positive correlation of fat with temperature suggests a constraint (birds becoming leaner under harsher conditions), while a negative correlation indicated a coping strategy (birds becoming fatter under harsher conditions).

In contrast to findings in other lowland species (Gosler 2002; Pilastro et al. 1995), we found for snowfinches a positive correlation between temperature and body mass in winter (Figure 4A). This result suggests that during winter snowfinches are constrained by environmental conditions in reaching their target body masses. The observation of lower body masses in colder conditions indicates that the snowfinch's energy balance may become negative at low temperatures, even when anthropogenic food is provided quasi ad libitum. This suggests that individuals visiting artificial feeding sites may still be unable to fully compensate for their energy expenditures. Compensation appears to occur during more favourable conditions when temperatures are higher.

Dynamics of body mass gain as a response to changed temperature conditions varied between winter and early spring. If a short‐term response to temperature was the main mechanism to regulate body mass, the pattern should be identical across seasons. Instead, seasonally different patterns suggest that fat accumulation is mainly regulated by distinct life cycle stages. This further supports our hypothesis that a biological clock with a circannual rhythm is key to regulating fat accumulation in this high‐elevation specialist. Differences in the relative importance of regulatory mechanisms may explain the contrasting observations between lowland and mountain species. In high‐elevation specialists, mitigating sudden temperature drops and prolonged periods of limited food availability during adverse weather likely requires more preparatory regulation of fat accumulation.

The positive correlation between average body mass and temperature suggests that, under cold conditions, snowfinches must take measures to counteract further reductions in fat reserves. An initial reduction in body mass possibly even triggers mitigation measures, such as physiological and behavioural changes. The observed opportunistic movements and short‐distance migration of snowfinches in winter (Bettega et al. 2020; Resano‐Mayor et al. 2020) likely reflect behavioural strategies as a response to deteriorated conditions. Vertical movements might act as short‐term responses to adverse conditions at high elevations. This is consistent with the GPS data from the tagged individuals in this study, which showed lower elevations during winter compared to the rest of the year. Temporarily visiting sites at lower elevations with milder climate is likely an immediate strategy to cope with colder temperatures at high elevations.

The body mass‐temperature relationship at feeding sites may be buffered by the anthropogenic food supply. Dynamics may differ for individuals that potentially avoid artificial feeding sites. For individuals relying primarily on natural food sources, temperature effects on body mass could be even stronger than observed in this study, as higher energy demands in colder conditions may coincide with lower food availability.

However, this study uncovered constrained body mass gain for both sexes in individuals that had access to bird feeders. It has been shown previously that, under specific environmental conditions, female snowfinches had lower apparent annual survivals compared to males (Strinella et al. 2020). Our results did not reveal morphological/physiological differences that could explain sex‐specific survival rates in winter or early spring. However, behavioural differences between the sexes observed in this study may contribute to sex‐specific survival in winter. In early spring we did not find constrained body mass gain rates for either sex. This suggests that mortality risk may be lower for both sexes during this period. Nonetheless, we cannot exclude the possibility that a cold spell in spring could have a more detrimental effect on survival than a similar event in winter, particularly given that absolute fat reserves are smaller in spring. Higher energy demands during a cold spell, combined with other factors, such as depleted natural food resources, cessation of food supply at bird feeders, and increased mating‐related energy expenditure, could elevate mortality risk during this period.

Our results suggest that long‐term temperature serves as an ultimate cue for the regulation of fat reserves in snowfinches. Ongoing climate warming, with generally higher temperatures, thus has the potential to decrease winter and spring fat reserves. While average temperatures have risen, the frequency of extreme events has increased in recent decades (Seneviratne et al. 2021). In the coming decades, individuals with lower fat reserves may face adverse weather conditions more frequently. Future studies should investigate how climate warming and extreme events affect seasonal and short‐term fat dynamics and survival. Crucial for predicting future population trends is clarifying whether climate change drives directed adaptations, such as reduced fat and body mass, and whether these adaptations are associated with altered demographic rates.

## 5 Conclusion

In line with our first hypothesis, snowfinches exhibited seasonal phenotypic flexibility in behavioural, morphological, and physiological traits. Movement patterns and home ranges were highly comparable among the six males but different for the two females (i.e., larger home range and short‐distance migration). This pattern aligns with our second hypothesis regarding sex‐specific phenotypic flexibility in movements. In contrast, seasonal dynamics of fat accumulation were similar between the sexes, contradicting our second hypothesis of sex‐specific patterns in this trait on a seasonal basis.

Consistent with our third hypothesis, dynamics of body mass changes in response to temperature conditions depended on the season. This suggests that distinct life cycle stages exist and that target body masses are mainly regulated in a circannual manner. The fact that seasonal fat accumulation aligned more closely with long‐term temperature trends than with temperature during the study period suggests that physiological adaptations do not keep pace with accelerated climatic changes.

Contrary to our fourth hypothesis, short‐term dynamics of fat accumulation did not differ markedly between females and males. Our results indicate that fat accumulation is constrained for both sexes during winter, but unconstrained in early spring. This pattern suggests that the morphological and physiological characteristics of both sexes may predispose them to higher mortality during winter. In contrast, the unconstrained fat gain in early spring implies that physiological capacities to cope with adverse conditions are greater at that time of year.

Taken together, the extent of fat reserves and the regulation of fat accumulation differ substantially from what was found in lowland passerine species, which we interpret as a physiological adaptation to the high‐elevation habitat. Beyond physiological adaptations, seasonal phenotypic flexibility in behaviour likely plays a critical role in survival at high elevations. Our results suggest that sex‐specific behaviour and behavioural flexibility are key adaptations for enduring winter conditions in high‐mountain environments.

## Author Contributions

**Sebastian Dirren:** conceptualization (equal), data curation (lead), formal analysis (equal), writing – original draft (lead), writing – review and editing (lead). **Carole Niffenegger:** conceptualization (equal), formal analysis (equal), writing – original draft (supporting), writing – review and editing (supporting). **Fränzi Korner‐Nievergelt:** conceptualization (equal), formal analysis (equal), project administration (lead), writing – original draft (supporting), writing – review and editing (supporting).

## Funding

This study was financially supported by SNF (project number 10.002.489), the Yvonne Jacob Foundation, Swarovski, and an anonymous foundation.

## Ethics Statement

This study complies with Swiss legal requirements for the capture, handling, saliva sampling, and tagging of snowfinches (authorisations: LU02/2016 27568, LU02/2019 31029 and LU02/2022 35285; capture and ringing permits issued by the Federal Office for the Environment).

## Conflicts of Interest

The authors declare no conflicts of interest.

## Supporting information


**Figure S1:** Long‐term average temperatures (1991–2020) are shown separately for the weather stations Segl Maria (Long‐term Temp. SIA) and Weissfluhjoch (Long‐term Temp. WFJ), as well as their combined mean (Long‐term Temp. SIA/WFJ). Mean temperatures for the study period are shown as the combined means for the weather stations Andermatt/Titlis (Mean Temp. 2017–2024 ANT/TIT) and Segl Maria/Weissfluhjoch (Mean Temp. 2017–2024 SIA/WFJ). Additionally, day length is plotted as monthly means.

**Figure S2:** (A–H) Home ranges of eight snowfinches calculated using kernel density estimates (colour lines: 95% contours). Colours correspond to individuals shown in (I). Capturing sites where food was constantly supplied during the winter months are shown as dark grey points, and Swiss buildings are shown in black. The female in (F) occupied two regions ~180 km apart. Home ranges were calculated separately for these two clusters of locations. Symbols indicate locations recorded in winter (diamonds) or other seasons (points). Highlighted symbols with yellow outlines show locations within a 50 m radius of buildings, and those with magenta outlines indicate locations within a 50 m radius of study sites. I. Violin plots showing elevational distributions of the eight individuals. Hillshade map and buildings layer swisstopo.

**Figure S3:** Residual plot of the linear mixed‐effects model with body mass as the response variable.

**Figure S4:** Residual plot of the linear model used to convert fat and muscle scores into fat and muscle masses.

**Figure S5:** Residual plot of the linear mixed‐effects model with fat mass as the response variable.

**Figure S6:** Body parts, including lean body mass, across sexes and months. Stacked bars show the absolute mass (g) of each body part, modelled using linear mixed‐effects models. Numbers inside the bars indicate each body part's mass as a percentage of total body mass.

**Figure S7:** Residual plot of the linear mixed‐effects model with muscle mass as the response variable.

**Figure S8:** Residual plot of the Bayesian linear mixed‐effects model with body mass as the response variable.

**Table S1:** Study sites with elevation, first captures, and respective recaptures. Bold: complete datasets containing body mass, wing chord, fat score, muscle score and sex; (total captures and recaptures). Numbers above the dashed line correspond to the winter dataset, below the dashed line to the summer dataset.
**Table S2:** Monthly numbers and percentages (in brackets) of genetically sexed females and males captured across all study sites. Numbers refer to successfully sexed individuals only.
**Table S3:** Statistics of elevational distributions and home ranges calculated as kerne density estimates (95% utilisation) of eight individuals.
**Table S4:** Parameter estimates from the linear mixed‐effects model, with body mass as the response variable.
**Table S5:** Parameter estimates from the linear model used to convert fat and muscle scores into corresponding fat and muscle masses.
**Table S6:** Parameter estimates from the linear mixed‐effects model, with fat mass as the response variable.
**Table S7:** Parameter estimates from the linear mixed‐effects model, with muscle mass as the response variable.
**Table S8:** Parameter estimates from the Bayesian linear mixed‐effects model, with body mass as the response variable.

## Data Availability

R codes and data are available on vogelwarte.ch Open Repository and Archive (https://doi.org/10.5281/zenodo.17425533).
