# Supplementary figures and images for: Beyond Circannual Fattening: Behavioural Flexibility and Sex‐Specific Strategies Enable Coping With High‐Elevation Winters
Source: Ecol Evol. 2026 Apr 17;16(4):e73482. doi: 10.1002/ece3.73482 (PMC13090115; doi:10.1002/ece3.73482)

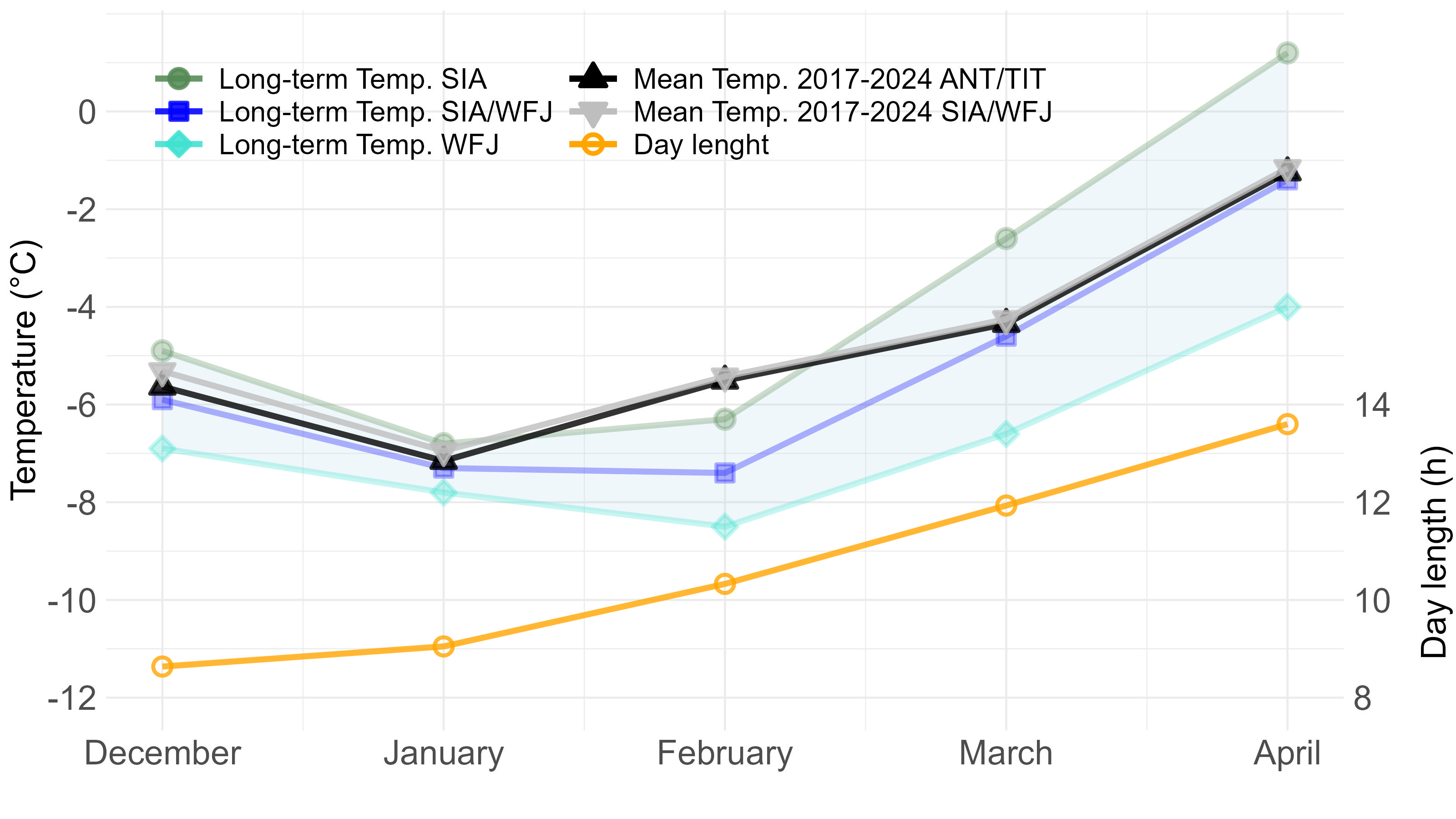

Supplement: Supplementary file 1 — Figure S1: Long‐term average temperatures (1991–2020) are shown separately for the weather stations Segl Maria (Long‐term Temp. SIA) and Weissfluhjoch (Long‐term Temp. WFJ), as well as their combined mean (Long‐term Temp. SIA/WFJ). Mean temperatures for the study period are shown as the combined means for the weather stations Andermatt/Titlis (Mean Temp. 2017–2024 ANT/TIT) and Segl Maria/Weissfluhjoch (Mean Temp. 2017–2024 SIA/WFJ). Additionally, day length is plotted as monthly means. [file ECE3-16-e73482-s005.jpeg]

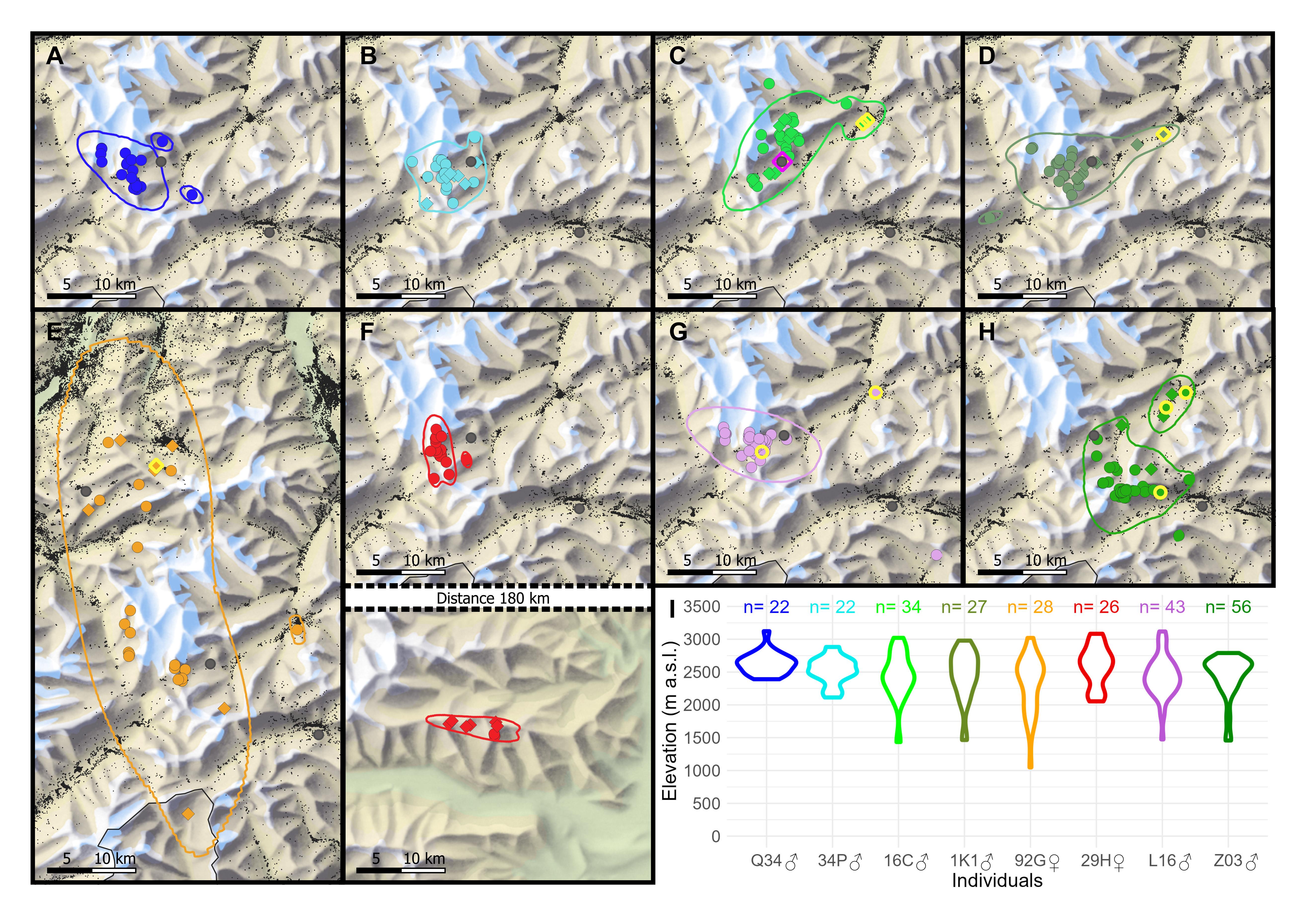

Supplement: Supplementary file 2 — Figure S2: (A–H) Home ranges of eight snowfinches calculated using kernel density estimates (colour lines: 95% contours). Colours correspond to individuals shown in (I). Capturing sites where food was constantly supplied during the winter months are shown as dark grey points, and Swiss buildings are shown in black. The female in (F) occupied two regions ~180 km apart. Home ranges were calculated separately for these two clusters of locations. Symbols indicate locations recorded in winter (diamonds) or other seasons (points). Highlighted symbols with yellow outlines show locations within a 50 m radius of buildings, and those with magenta outlines indicate locations within a 50 m radius of study sites. I. Violin plots showing elevational distributions of the eight individuals. Hillshade map and buildings layer swisstopo. [file ECE3-16-e73482-s008.jpeg]

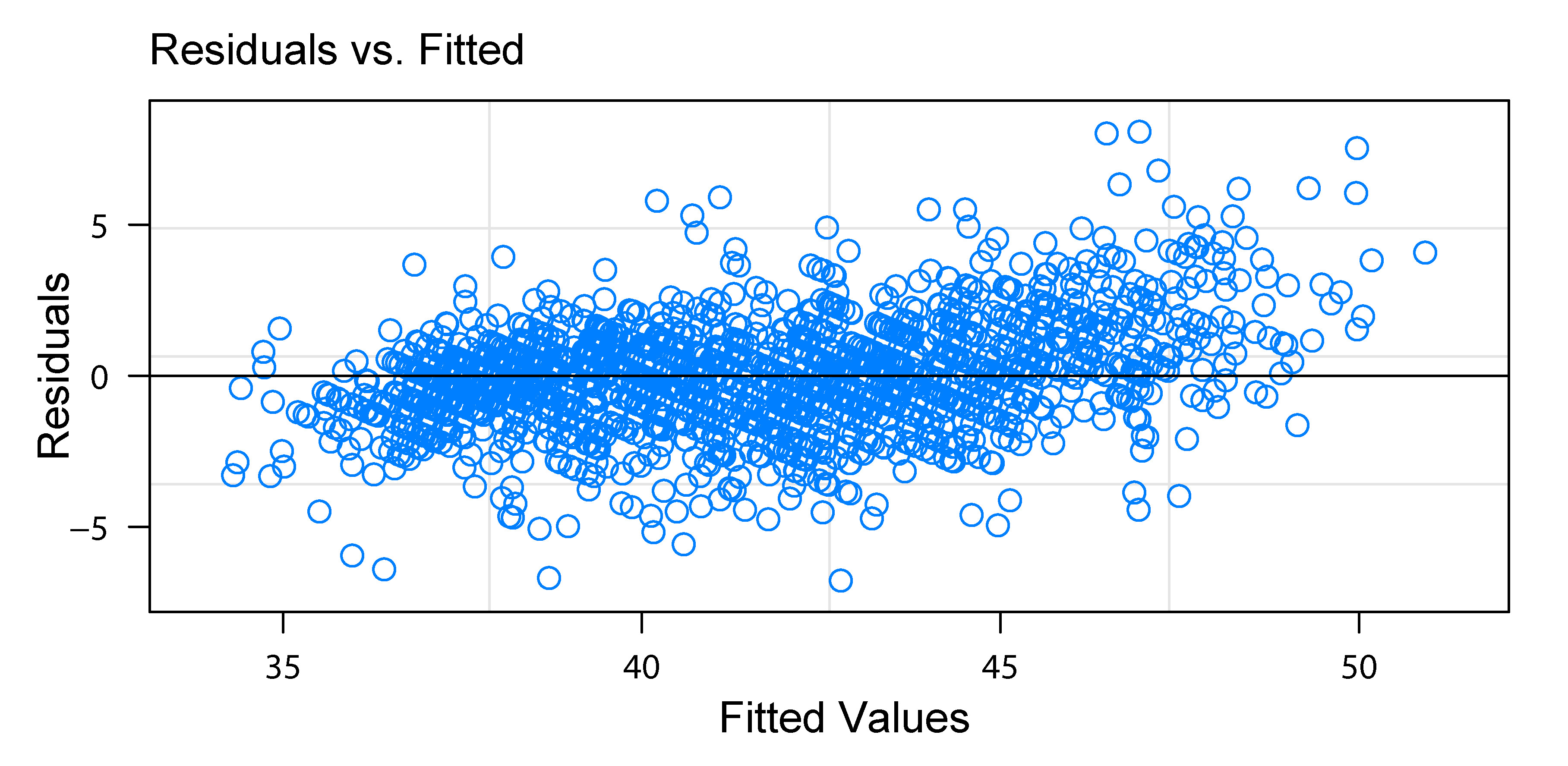

Supplement: Supplementary file 3 — Figure S3: Residual plot of the linear mixed‐effects model with body mass as the response variable. [file ECE3-16-e73482-s003.jpeg]

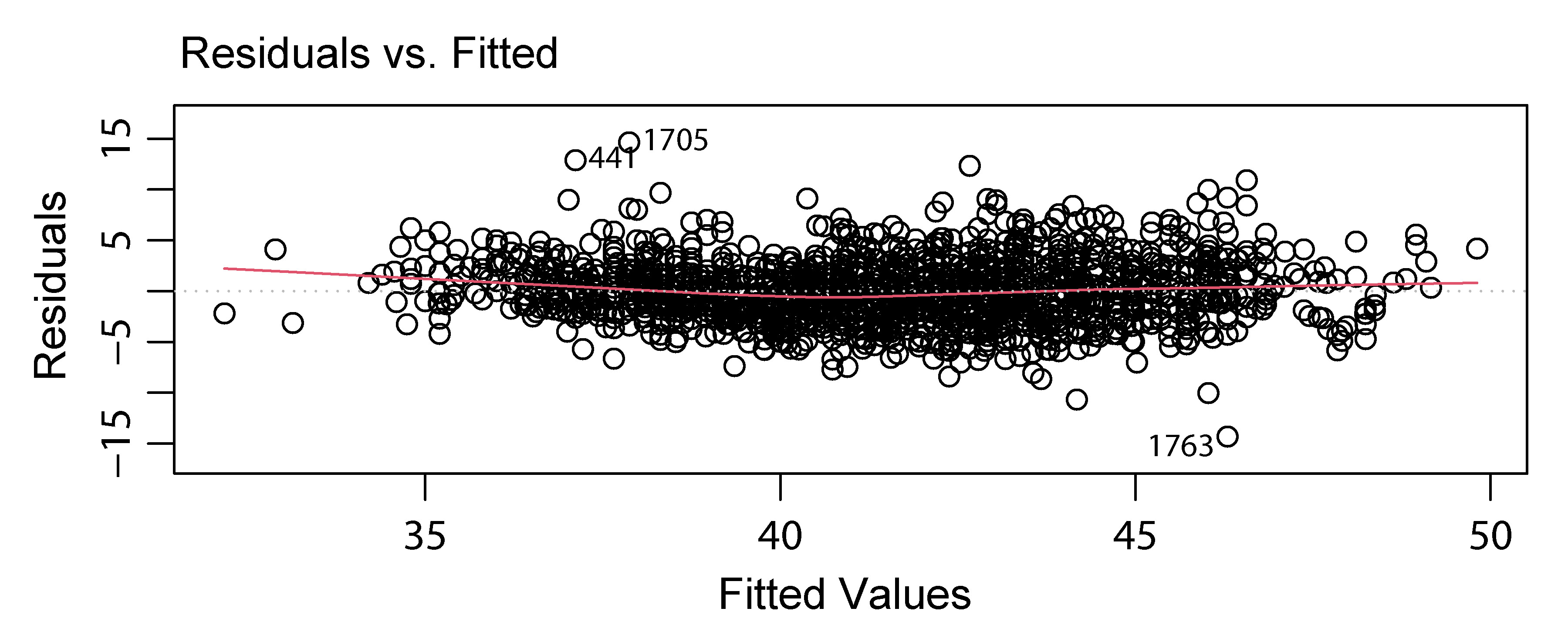

Supplement: Supplementary file 4 — Figure S4: Residual plot of the linear model used to convert fat and muscle scores into fat and muscle masses. [file ECE3-16-e73482-s001.jpeg]

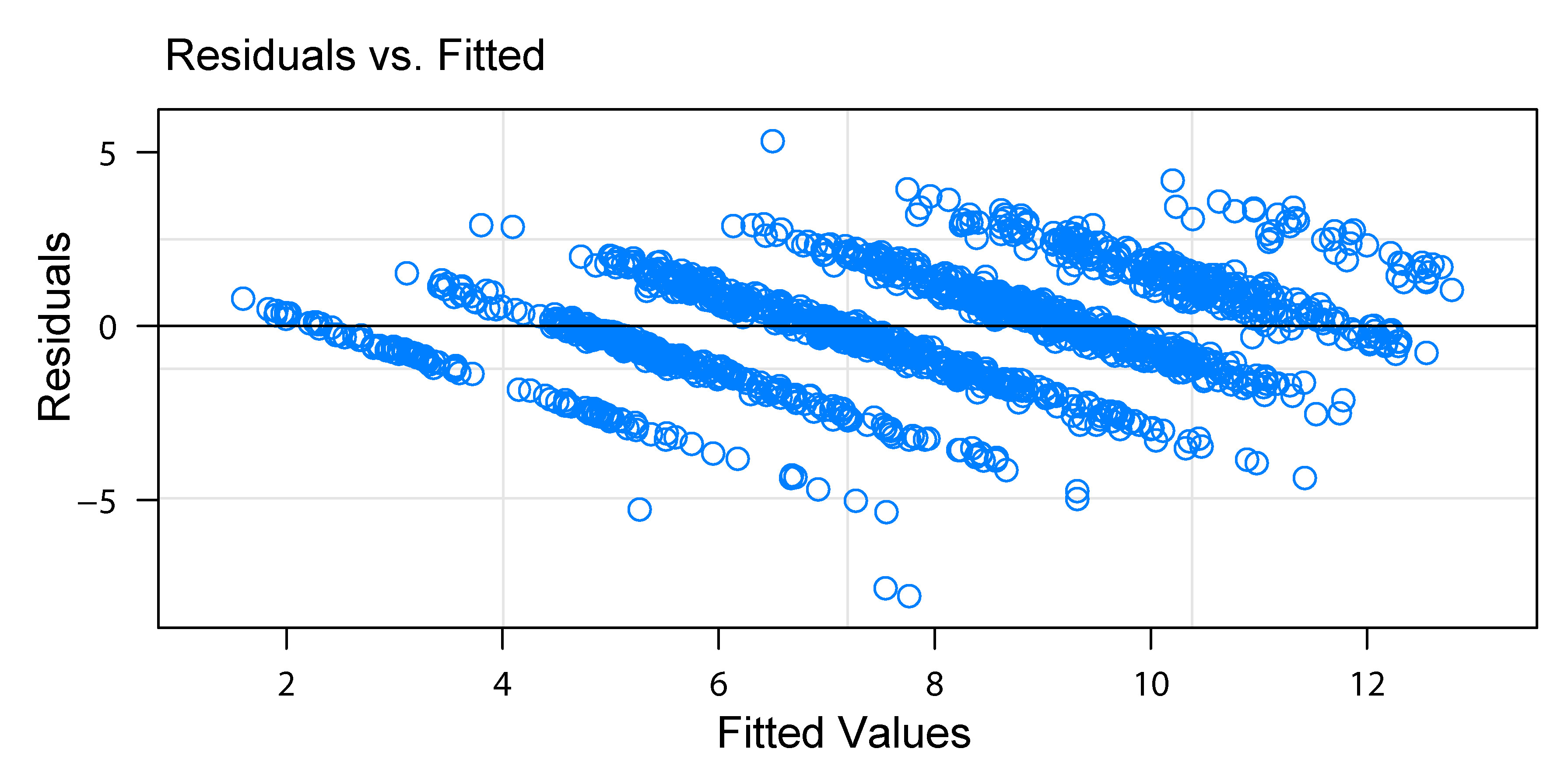

Supplement: Supplementary file 5 — Figure S5: Residual plot of the linear mixed‐effects model with fat mass as the response variable. [file ECE3-16-e73482-s006.jpeg]

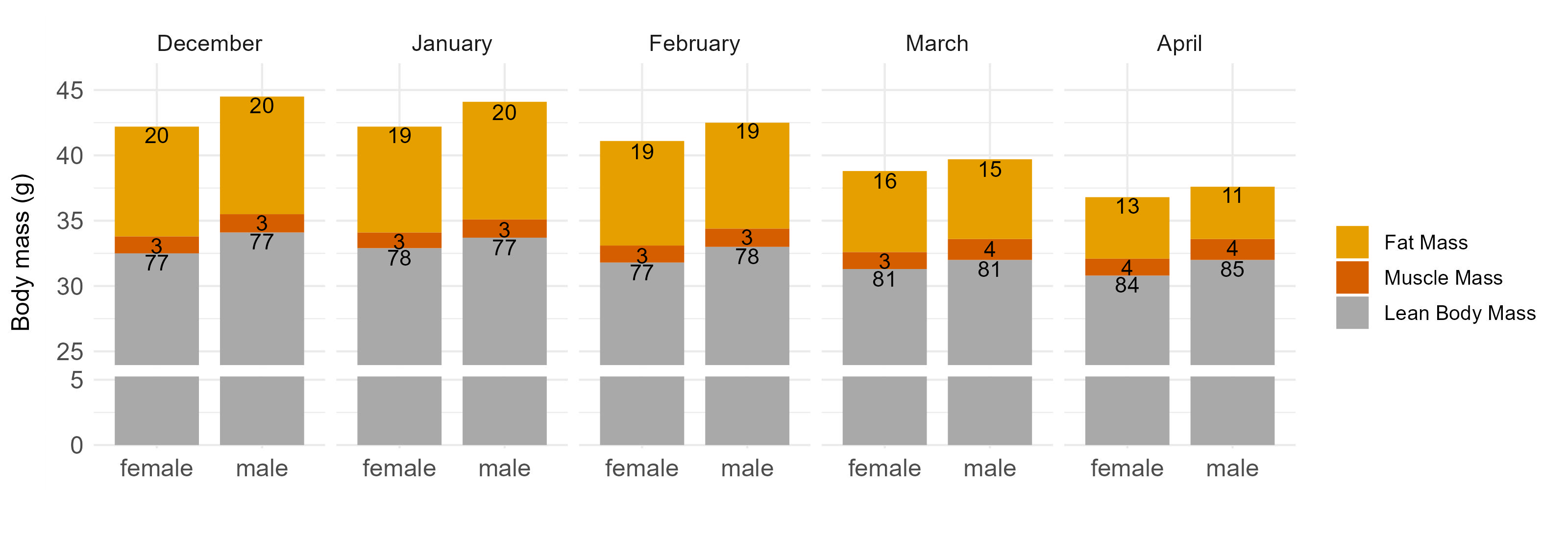

Supplement: Supplementary file 6 — Figure S6: Body parts, including lean body mass, across sexes and months. Stacked bars show the absolute mass (g) of each body part, modelled using linear mixed‐effects models. Numbers inside the bars indicate each body part's mass as a percentage of total body mass. [file ECE3-16-e73482-s007.jpeg]

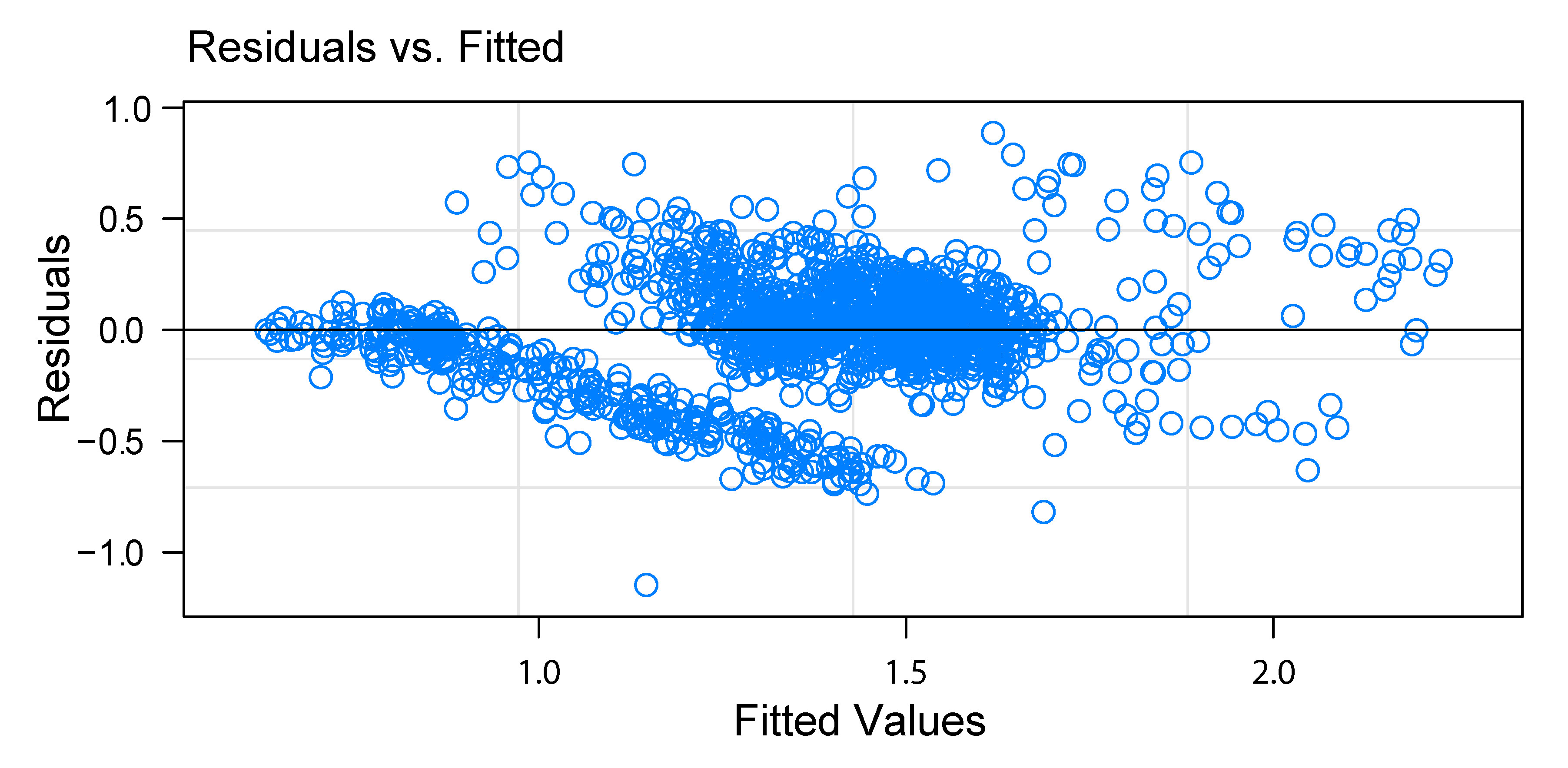

Supplement: Supplementary file 7 — Figure S7: Residual plot of the linear mixed‐effects model with muscle mass as the response variable. [file ECE3-16-e73482-s004.jpeg]

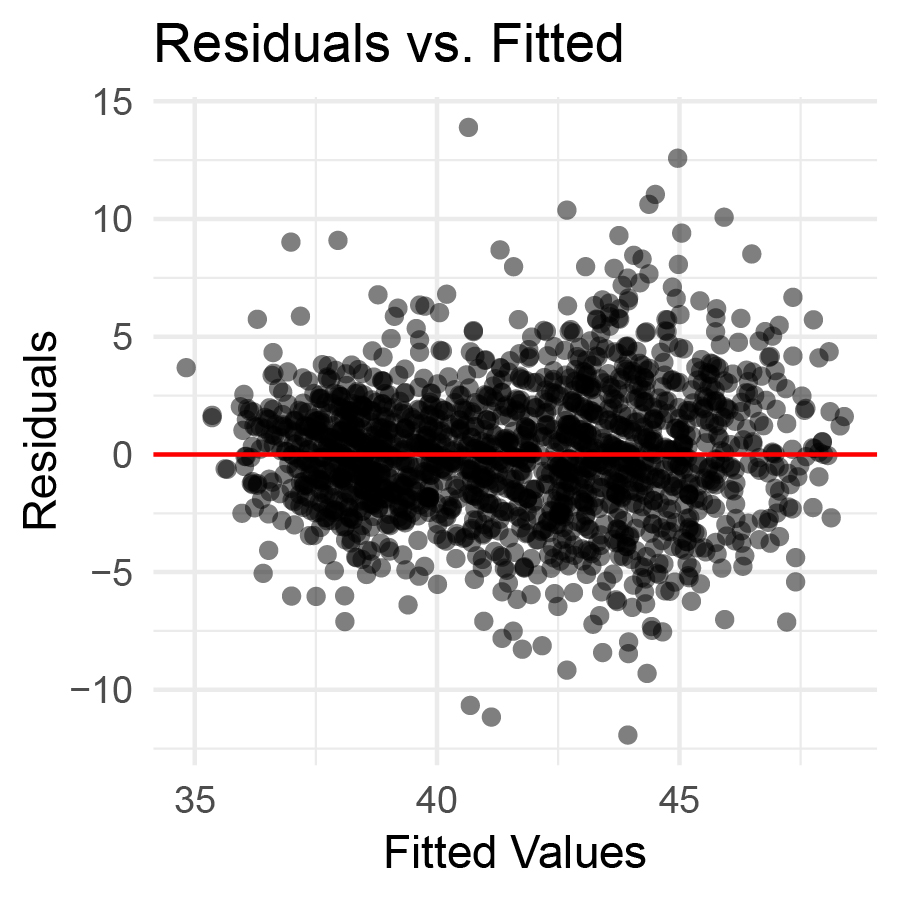

Supplement: Supplementary file 8 — Figure S8: Residual plot of the Bayesian linear mixed‐effects model with body mass as the response variable. [file ECE3-16-e73482-s009.jpeg]
