# Supplementary material for: Beyond Circannual Fattening: Behavioural Flexibility and Sex‐Specific Strategies Enable Coping With High‐Elevation Winters
Source: Ecol Evol. 2026 Apr 17;16(4):e73482. doi: 10.1002/ece3.73482 (PMC13090115; doi:10.1002/ece3.73482)
Supplement: Supplementary file 9 — Table S1: Study sites with elevation, first captures, and respective recaptures. Bold: complete datasets containing body mass, wing chord, fat score, muscle score and sex; (total captures and recaptures). Numbers above the dashed line correspond to the winter dataset, below the dashed line to the summer dataset. Table S2: Monthly numbers and percentages (in brackets) of genetically sexed females and males captured across all study sites. Numbers refer to successfully sexed individuals only. Table S3: Statistics of elevational distributions and home ranges calculated as kerne density estimates (95% utilisation) of eight individuals. Table S4: Parameter estimates from the linear mixed‐effects model, with body mass as the response variable. Table S5: Parameter estimates from the linear model used to convert fat and muscle scores into corresponding fat and muscle masses. Table S6: Parameter estimates from the linear mixed‐effects model, with fat mass as the response variable. Table S7: Parameter estimates from the linear mixed‐effects model, with muscle mass as the response variable. Table S8: Parameter estimates from the Bayesian linear mixed‐effects model, with body mass as the response variable. [file ECE3-16-e73482-s002.docx]

Table S1. Study sites with elevation, first captures, and respective recaptures. Bold: complete datasets containing body mass, wing chord, fat score, muscle score and sex; (total captures and recaptures). Numbers above the dashed line correspond to the winter dataset, below the dashed line to the summer dataset.

| Location | Elevation (m a.s.l.) | First captures | Recaptures | total |
| --- | --- | --- | --- | --- |
| Airolo TI | 1426 | **112** (162) | **6** (15) | 118 (177) |
| Arosa GR | 1900 | **186** (199) | 18 (23) | 204 (222) |
| Glarus Süd GL | 824 | **9** (11) | 0 (0) | 9 (11) |
| Kerns OW | 1900 | **327** (383) | 86 (105) | 413 (488) |
| Orsières VS | 1612 | **0** (12) | 0 (1) | 0 (13) |
| Realp UR | 2102 | **380** (488) | 201 (277) | 581 (765) |
| Scuol GR | 2057 | **5** (26) | 0 (1) | 5 (27) |
| St. Moritz GR | 2483 | 321 (403) | 23 (36) | 344 (439) |
| Surses GR | 1976 | 16 (22) | 0 (0) | 16 (22) |
|  | total winter | 1356 (1706) | 334 (458) | 1690 (2164) |
| Realp UR | 2430 | 2 (5) | 5 (10) | 7 (15) |

Table S2. Monthly numbers and percentages (in brackets) of genetically sexed females and males captured across all study sites. Numbers refer to successfully sexed individuals only.

| Month | females | males | n |
| --- | --- | --- | --- |
| December | 35 (**22.3**) | 122 (**77.7**) | 157 |
| January | 103 (25.2) | 305 (74.8) | 408 |
| February | 106 (21.5) | 388 (78.5) | 494 |
| March | 116 (26.9) | 316 (73.1) | 432 |
| April | 118 (33.1) | 239 (66.9) | 357 |

Table S3. Statistics of elevational distributions and home ranges calculated as Kerne Density Estimates (95% utilisation) of eight individuals.

| Individual | Elevations | | | Kernel Density Estimates | | |  |
| --- | --- | --- | --- | --- | --- | --- | --- |
|  | minimal elev. (m a.s.l.) | mean elev. (m a.s.l.) | maximal elev. (m a.s.l.) | low (km^2^) | est (km^2^) | high (km^2^) | n |
| 29H ♀ | 2054 | 2591 | 3083 | 22 | 43 | 72 | 26 |
| 34P ♂ | 2113 | 2520 | 2882 | 36 | 58 | 85 | 22 |
| Q34 ♂ | 2391 | 2635 | 3122 | 40 | 65 | 96 | 22 |
| L16 ♂ | 1474 | 2433 | 3118 | 67 | 92 | 122 | 43 |
| 1K1 ♂ | 1465 | 2453 | 2979 | 70 | 108 | 153 | 27 |
| 16C ♂ | 1434 | 2439 | 3021 | 83 | 120 | 165 | 34 |
| Z03 ♂ | 1458 | 2428 | 2790 | 94 | 125 | 160 | 56 |
| 92G ♀ | 1048 | 2322 | 3020 | 551 | 836 | 1179 | 28 |

Table S4. Parameter estimates from the linear mixed-effects model, with body mass as the response variable.

Variance explained by the model: marginal R^2^ = 0.36; conditional R^2^ = 0.81 (Nakagawa & Schielzeth, 2013).

Fixed effects

| Effect | Estimate | Std. Error |
| --- | --- | --- |
| Intercept | 40.514 | 1.053 |
| Month1 | 0.990 | 1.128 |
| Month2 | -0.910 | 1.134 |
| Month3 | -1.821 | 1.192 |
| Month4 | -3.814 | 1.212 |
| Sex (male) | 2.284 | 0.616 |
| Time since sunrise | 0.430 | 0.192 |
| Month1:Sex (male) | -0.566 | 0.666 |
| Month2:Sex (male) | -0.761 | 0.662 |
| Month3:Sex (male) | -1.428 | 0.665 |
| Month4:Sex (male) | -1.485 | 0.678 |
| Sex (male):Time since sunrise | 0.009 | 0.067 |
| Month1:Time since sunrise | -0.242 | 0.208 |
| Month2:Time since sunrise | -0.074 | 0.199 |
| Month3:Time since sunrise | -0.406 | 0.213 |
| Month4:Time since sunrise | -0.393 | 0.199 |

Random effects (Std. Dev.)

| Group | Std. Dev. |
| --- | --- |
| Individual | 1.903 |
| Location/Day | 1.751 |
| Location | 0.384 |
| Year | 0.584 |
| Residual | 2.261 |

Table S5. Parameter estimates from the linear model used to convert fat and muscle scores into corresponding fat and muscle masses.

Total variance explained by the model: R^2^ = 0.49.

| Effect | Estimate | Std. Error |
| --- | --- | --- |
| Intercept | 15.446 | 13.618 |
| Wing chord | 0.139 | 0.111 |
| Fat score | 0.263 | 2.062 |
| Muscle score | -2.052 | 5.750 |
| Wing chord × Muscle score | 0.023 | 0.047 |
| Wing chord × Fat score | 0.017 | 0.017 |

Table S6. Parameter estimates from the linear mixed-effects model, with fat mass as the response variable.

Variance explained by the model: marginal R^2^ =0.33; conditional R^2^ = 0.76 (Nakagawa & Schielzeth, 2013).

Fixed effects

| Effect | Estimate | Std. Error |
| --- | --- | --- |
| Intercept | 7.784 | 0.822 |
| Month1 | -0.083 | 0.751 |
| Month2 | -0.575 | 0.757 |
| Month3 | -1.921 | 0.800 |
| Month4 | -2.777 | 0.818 |
| Sex (male) | 0.430 | 0.370 |
| Time since sunrise | 0.167 | 0.123 |
| Month1:Sex (male) | 0.243 | 0.399 |
| Month2:Sex (male) | -0.447 | 0.400 |
| Month3:Sex (male) | -0.664 | 0.402 |
| Month4:Sex (male) | -1.262 | 0.415 |
| Sex (male):Time since sunrise | 0.040 | 0.042 |
| Month1:Time since sunrise | -0.055 | 0.132 |
| Month2:Time since sunrise | 0.031 | 0.128 |
| Month3:Time since sunrise | -0.086 | 0.136 |
| Month4:Time since sunrise | -0.237 | 0.127 |

Random effects (Std. Dev.)

| Group | Std. Dev. |
| --- | --- |
| Individual | 0.586 |
| Location/Day | 1.257 |
| Ringer | 1.017 |
| Location | 0.000054 |
| Year | 0.981 |
| Residual | 1.610 |

Table S7. Parameter estimates from the linear mixed-effects model, with muscle mass as the response variable.

Variance explained by the model: marginal R^2^ = 0.1; conditional R^2^ = 0.64 (Nakagawa & Schielzeth, 2013).

Fixed effects

| Effect | Estimate | Std. Error |
| --- | --- | --- |
| Intercept | 1.397 | 0.126 |
| Month1 | -0.170 | 0.098 |
| Month2 | -0.213 | 0.099 |
| Month3 | -0.165 | 0.105 |
| Month4 | -0.129 | 0.108 |
| Sex (male) | 0.138 | 0.056 |
| Time since sunrise | -0.028 | 0.017 |
| Month1:Sex (male) | 0.025 | 0.061 |
| Month2:Sex (male) | 0.060 | 0.061 |
| Month3:Sex (male) | 0.110 | 0.061 |
| Month4:Sex (male) | 0.201 | 0.063 |
| Sex (male):Time since sunrise | -0.007 | 0.006 |
| Month1:Time since sunrise | 0.028 | 0.019 |
| Month2:Time since sunrise | 0.051 | 0.018 |
| Month3:Time since sunrise | 0.055 | 0.019 |
| Month4:Time since sunrise | 0.038 | 0.018 |

Random effects (Std. Dev.)

| Group | Std. Dev. |
| --- | --- |
| Individual | 0.080 |
| Location/Day | 0.135 |
| Ringer | 0.071 |
| Location | 0.041 |
| Year | 0.222 |
| Residual | 0.249 |

Table S8. Parameter estimates from the Bayesian linear mixed-effects model, with body mass as the response variable.

The leave-one-out adjusted R^2^ value of the model was 0.48.

Fixed effects

| Effect | Estimate | Est. Error | 95% CI (lower) | 95% CI (upper) |
| --- | --- | --- | --- | --- |
| Intercept | 21.074 | 3.531 | 14.213 | 28.009 |
| Temperature | 0.141 | 0.091 | -0.037 | 0.319 |
| Month.1 | -0.495 | 0.776 | -2.003 | 1.057 |
| Month.2 | -2.109 | 0.740 | -3.633 | -0.685 |
| Month.3 | -5.838 | 1.043 | -7.879 | -3.740 |
| Month.4 | -7.929 | 1.178 | -10.276 | -5.544 |
| Wing | 0.183 | 0.029 | 0.127 | 0.240 |
| Sex (male) | 0.815 | 0.509 | -0.208 | 1.807 |
| Time since sunrise | 0.218 | 0.071 | 0.078 | 0.351 |
| Sex (male): Time since sunrise | -0.041 | 0.075 | -0.202 | 0.103 |
| Temperature:Sex (male) | -0.014 | 0.060 | -0.132 | 0.099 |
| Temperature:Season (Mar-Apr) | -0.290 | 0.140 | -0.559 | -0.019 |
| Sex (male):Season (Mar-Apr) | -0.276 | 0.622 | -1.541 | 0.919 |
| Temperature:Sex (male):Season (Mar-Apr) | 0.073 | 0.096 | -0.122 | 0.260 |

Random effects

| Group | Estimate | Est. Error | 95% CI (lower) | 95% CI (upper) |
| --- | --- | --- | --- | --- |
| Location | 0.801 | 0.667 | 0.034 | 2.656 |
| Location/Day | 1.661 | 0.182 | 1.332 | 2.041 |
| Year | 0.896 | 0.532 | 0.095 | 2.179 |

Reference

Nakagawa, S., and H. Schielzeth. 2013. “A General and Simple Method for Obtaining R^2^ From Generalized Linear Mixed‐Effects Models.” *Methods in Ecology and Evolution* 4, no. 2: 133–142. https://doi.org/10.1111/j.2041-210x.2012.00261.x.
